# Continuous dynamic adjustment of the plant circadian oscillator

**DOI:** 10.1038/s41467-019-08398-5

**Published:** 2019-02-01

**Authors:** Alex A. R. Webb, Motohide Seki, Akiko Satake, Camila Caldana

**Affiliations:** 1 Department of Plant Sciences, Downing Street, Cambridge, CB3 0LJ UK; 2 Faculty of Design, Kyushu University, 4-9-1 Shiobaru, Minamiku, Fukuoka, 815-8540 Japan; 3 Department of Biology, Faculty of Science, Kyushu University, Fukuoka, 819-0395 Japan; 4 Max Planck Institute of Molecular Plant Physiology, Am Mühlenberg 1, 14476 Potsdam-Golm, Germany

## Abstract

The clockwork of plant circadian oscillators has been resolved through investigations in *Arabidopsis thaliana*. The circadian oscillator is an important regulator of much of plant physiology, though many of the mechanisms are unclear. New findings demonstrate that the oscillator adjusts phase and period in response to abiotic and biotic signals, providing insight in to how the plant circadian oscillator integrates with the biology of the cell and entrains to light, dark and temperature cycles. We propose that the plant circadian oscillator is dynamically plastic, in constant adjustment, rather than being an isolated clock impervious to cellular events.

## Abstract (web summary)

Biological circadian rhythms maintain a period close to 24 h in coordination with the Earth’s fixed rotational period. Here Webb et al. discuss how external cues continuously adjust phase and period, viewing the oscillator as a dynamically-adjusted plastic system rather than tightly-coupled cogs in a mechanical clock.

## Introduction

### Circadian clocks do not have a fixed period

**Fig. 1:**
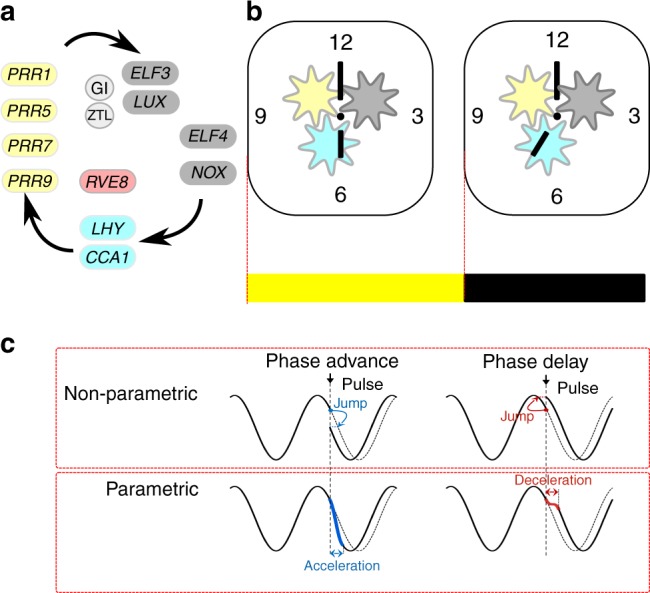
Circadian oscillators are entrained timekeepers. **a** The circadian oscillator contains a network of transcriptional regulators. The components regulate each other in a temporal series, indicated by the flow of the arrows. The extensive regulation between components has been omitted for clarity; for details, see references in the text. The components can be grouped functionally as MYB-like repressors (cyan), MYB-like activators (red), pseudo response regulators (yellow), nocturnal regulators (dark grey) and proteins involved in protein stability (light grey). **b** The circadian oscillator is often conceptualised as a mechanical clock, with “cogs” made up of the functional groups of transcriptional regulators, “hands” that provide a read-out of time and the clock is set to a new time at light (yellow box) and dark (black box) interfaces (red dotted line). **c** Entrainment is thought to occur through non-parametric changes that jump from one point in the cycle to another in an almost instantaneous change in state of the oscillator and parametric changes that require acceleration or deceleration of the oscillator

Circadian clocks drive biological rhythms to anticipate the light, dark and temperature cycles of a rotating planet, and to phase and sequence biological events in a coordinated manner. Circadian systems also modulate signalling pathways such that the biological response to a stimulus can depend on the time of day that the stimulus is delivered^1–6^. The rhythm-generating circadian oscillators have some remarkable properties^7–9^: they are capable of self-sustained oscillations with a period close to 24 h in constant environmental conditions; they can entrain to forced external cycles through phase and period adjustment; just like a pendulum, the oscillator can have a natural frequency, which means that there is period of light and dark cycles (usually near 24 h) with which the oscillator resonates to produce the highest amplitude rhythms, and circadian oscillators are temperature compensated such that the period of the circadian oscillator is less sensitive to temperature in the physiological range than would be expected from physical laws. While these behaviours are not unique within biology to circadian oscillators^10^, their sum has tended to endow circadian oscillators an air of mystery, which can make them appear outside the realms of normal biology. These properties have resulted in circadian oscillators as often being referred to as “clocks” (Fig. 1). This analogy has gained traction with the discoveries that plant, animal and fungi circadian oscillators include negative feedback loops of transcriptional regulation, which sometimes are considered analogous to the cogs of a mechanical watch (Fig. 1). The analogy has power because the feedback in circadian oscillators, like the cogs of a watch, generate the timing signals, provide robustness and temperature compensation, and are the basis of adjusting to external time signals^11^. However, these “clocks” are not impervious to internal and external signals, they can respond to environmental signals through changes in period and phase. We discuss why circadian clocks respond to environmental signals by a change in period when the Earth has a fixed rotational period. We focus on the model plant *Arabidopsis thaliana* but consider the concepts might be broadly applicable to the circadian clocks of other plants, fungi, single-celled organisms and peripheral organs of mammals. The situation in cells where the molecular circadian oscillators are strongly coupled by electrical signalling, such as in the suprachiasmatic nucleus of mammalian brains, might be different because the strong coupling will render those oscillators less sensitive to perturbation.

### Dynamic adjustment of Arabidopsis circadian period

The time taken for the Arabidopsis circadian oscillator to complete one cycle in constant environmental conditions, known as the free-running period, is altered by light, temperature, metabolites such as sugars, hormones such as ethylene and ions such as Ca^2+^ (Table 1). Circadian period decreases with increased light intensity^12^, whereas it increases with longer photoperiods during entrainment^13^. Increased temperature reduces circadian period, though the period response to temperature is much less than most other biological activities, which have a rate of change of two to three in response to 10 °C alterations in the physiological range^14,15^. Low sugar availability increases circadian period, and if under these conditions sucrose, glucose or fructose are added back into the system, the circadian period decreases^16^. Other metabolites can also affect circadian period; 3′-phosphoadenosine 5′-phosphate and nicotinamide both increase circadian period^17,18^. Hormones can also affect circadian period, with ethylene reducing the period^19^ and abscisic acid (ABA) being reported to both increase^20^ and decrease^21^ the period. Additionally, ions can regulate the circadian period. The effect of Ca^2+^ is through signalling^22^, whereas that of Fe^3+^ could be nutritional^23^. Sugars and ABA regulate the circadian period rapidly through transcriptional networks suggesting that the signalling pathways that adjust the oscillator period have arisen to confer advantage^21,24^. Variability of the circadian period also occurs between the cells of a plant. Root cells have greater variation in circadian period than those in the hypocotyl and cotyledon, those at the top of the root have a longer period than the hypocotyl, but those in the root tip have a very fast period^25^.

**Table 1:** Signals that adjust the free-running period of the circadian oscillator of Arabidopsis

Stimulus	Effect on free-running circadian period	Molecular basis
Abscisic acid	Lengthen^20^ Shorten^21^	ABA induction of *TOC1* expression dependent on *MYB63*
Ethylene	Shorten^19^	*GI*-dependent, conditional on sucrose
Homobrassinolide	Shorten^20^	
Sucrose	Shorten^73^ Shorten, when endogenous sugars are depleted^16,24^ Shorten, when endogenous sugars are depleted^53^	*SFR6* Regulation of *PRR7* through downregulation of bZIP63 activity PIF binding to the CCA1 and LHY promoters
Glucose	Shorten, when endogenous sugars are depleted^16^	
Fructose	Shorten, when endogenous sugars are depleted^16^	
3′-Phosphoadenosine 5′-phosphate	Lengthen^17^	
Nicotinamide	Lengthen^18^ Lengthen^34^	Inhibition of ADPR cyclase activity and Ca^2+^ signalling Reduction of H3K4me3 accumulation
Ca^2+^	Interruption of Ca^2+^ signalling in the cytosol lengthens period^22^	Ca^2+^ sensed by CALMODULIN-LIKE24 regulates the oscillator through genetic interactions with *TOC1* and *CHE*
Fe^3+^	Shorten^23^	
Light intensity	Shorten^12^	Phytochrome and cryptochrome signalling
Total period length (T cycle)	Forced entrainment during T cycle, which is transient. No effect on free-running period^46^	
Increased photoperiod	Lengthen^13^	*BIG*
Osmotic stress (high mannitol)	Lengthen^17^	Blue-light dependent
High temperature	Shorten^14^ Shorten^15^	Regulation of SUMOylation

The ability of these signals to rapidly adjust the circadian period is an example of phenotypic plasticity in response to a stimulus, where the phenotype is circadian period. The phenotypic plasticity is dynamic because the degree and direction of the change in period can depend on the time of day and is reversible. For these reasons, we call the responses of circadian period to stimuli “dynamic plasticity”. This stimulus-regulated dynamic plasticity of the circadian period should not be confused with the effects of genetic mutations that alter the circadian period under all conditions, such as the laboratory isolated short- and long-period circadian mutants of Arabidopsis^8^.

Some of the signals that regulate the circadian period, including light and temperature (Table 1), are associated with the synchronisation of the circadian oscillator with the cycles of the environment in the process of entrainment. Circadian entrainment is the adjustment of the phase of the circadian oscillator to match the timing of light and temperature cycles so that the system can track the time of dawn as it changes through the seasons. The founders of modern circadian biology advanced two theories for the mechanisms of entrainment^26,27^: Pittendrigh proposed that the circadian oscillator is advanced every day in an almost instantaneous manner in a non-parametric view of entrainment, it is a discontinuous event that causes rapid changes in the state of the circadian oscillator (Fig. 1c). Whereas Aschoff considered entrainment as a continuous process in which the circadian oscillator constantly accelerates and decelerates to adapt to the environment in parametric entrainment (Fig. 1c). We argue that the dynamic plasticity of the circadian period provides an insight into the molecular mechanisms of entrainment and can contribute to understanding how both parametric and non-parametric entrainment might occur, insight that was not available to the proposers of these hypotheses. However, other signals that regulate the circadian period are not obviously associated with environmental cycles, e.g. ABA and ethylene (Table 1); therefore we consider how dynamic plasticity might also couple the oscillator to the biology of the cell.

### Dynamic plasticity of circadian period involves *PRR7*

**Fig. 2:**
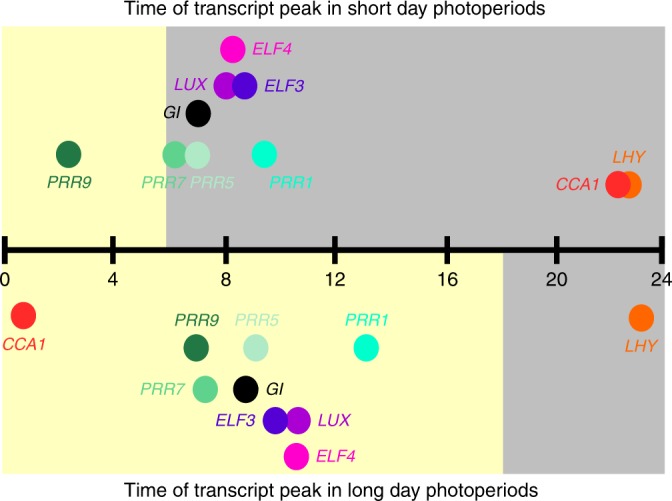
The loosely coupled nature of the oscillator is demonstrated by the plasticity of peak time of expression of the components of the circadian oscillator. Peak of oscillator transcript abundance is plotted against time since dawn (h). Upper plot: a photoperiod of 6 h light (yellow box) and 18 h dark (grey box); lower plot:18 h light 6 h dark. The time of the peaks shifts between the photoperiods relative to the external photoperiod and relative to each other. Distance from the time line on the *y* axis is for plotting clarity and conveys no meaning. The data were obtained from Table S2^45^

The network properties provide an insight into how the circadian oscillator can have dynamic plasticity of period. Temporal waves of transcriptional regulation result in the components of the oscillator having peak activity at different times of the day (Figs. 1 and 2). CIRCADIAN CLOCK ASSOCIATED 1 (CCA1) and the closely related LATE ELONGATED HYPOCOTYL (LHY) are MYB-like transcription factors that are expressed near dawn; these repress the expression of a suite of *PSEUDORESPONSE REGULATOR* (*PRR*) genes that are expressed in sequence (*9,7,5* and *1* (also known as *Timing of CAB expression 1* (*TOC1*))), with *PRR1* (*TOC1*) having the peak expression near dusk^8^. The PRRs repress *CCA1/LHY* expression^8^. Thus, once *PRR* expression has been activated by REVEILLE 8, another MYB-like protein^28^, the PRRs prevent the expression of *CCA1/LHY* until near the next dawn. In the night, there is targeting of TOC1 protein for degradation by the F-box protein ZEITLUPE (ZTL)^29^. GIGANTEA (GI) has a role in stabilising ZTL protein during the day and preventing its interaction with TOC1 proteins until night^30^. The evening complex forms by the co-binding of EARLY FLOWERING 3 (ELF3) and 4 (ELF4) with LUX ARRHYTHMO (LUX), which together might act as repressors in the circadian network^31^. LIGHT-REGULATED WD1 (LWD1) along with the members of the TEOSINTE BRANCHED 1-CYCLOIDEA-PCF (TCP) transcription factor family, including CCA1 HIKING EXPEDITION 1 (CHE) (TCP20), bind to the *CCA1* promoter to regulate its expression^32^. Light input to the system is provided by the phytochromes, acting through PHYTOCHROME INTERACTING (PIF) proteins^33^, cryptochromes and ZTL^8^. Post-translational modifications of the circadian oscillator components contribute to circadian timing through Ca^2+^ signalling^22^, histone modifications^34,35^, polyADPribosylation^36^ phosphorylation and protein translocation^37^.

Defects in the components of the oscillator can change the free-running period, or even result in arrhythmia. For example, *toc1-1* is a short period mutant^38^, *ztl-1* is a long period mutant^39^ and *elf3* plants become arrhythmic in constant light^40^. Some mutations in the circadian oscillator components also can affect the ability of the circadian oscillator to dynamically adjust circadian period in response to signals. For example, *prr7* mutants have a fixed circadian period in red light, whereas the period of the wild type shortens with increased light intensity^41^. The relatively invariant period of *prr7* mutants compared to the dynamic plasticity of the wild type has been demonstrated also for sucrose^16^ and nicotinamide^42^. Sugars such as glucose, fructose and sucrose shorten the circadian period^16^, while nicotinamide increases the circadian period^42^, but both these responses are absent in *prr7* mutants. Thus, while *prr7* mutants are usually described as long period^8^, this might be an incomplete description. Instead the apparent long period phenotype of *prr7* mutants might be the result of the relative dynamic plasticity of circadian period in the wild type, compared to the rigidity of period in *prr7* mutants. The inability of *prr7* mutants to dynamically adjust circadian period in response to red light, sugars and nicotinamide suggests that *PRR7* might have a function in regulating the period of the circadian oscillator. It has been proposed that PRR7, working alongside PRR9, acts as a toggle switch that drives the oscillator from the morning state of CCA1/LHY activity to the evening state of TOC1 activity^43^. This role of driver in a toggle switch from one bistable system to another might make PRR7 and PRR9 uniquely suited to receive inputs that adjust the circadian clock to environmental signals, and the pulse like expression of *PRR7/9* provides the ability to restrict the responses to particular times of the day^43^.

### Circadian phase is dynamically plastic

Like circadian period, circadian phase is also dynamically plastic. Phase refers to the relationship between an internal event and that of the external time. For example, some events in the circadian oscillator might have peak activity at dawn, or dusk. Typically, these are referred to as phased at dawn or dusk. Due to changes in the day length at higher latitudes, the timing of the events in the oscillator can phase adjust through the process of entrainment to ensure coincidence between internal timing and events in the environment. For example, in Cambridge, United Kingdom, Latitude 52° N, the time of dawn changes by about 4 h over the year due to seasonal changes. For *CCA1* expression to peak near dawn, the time of the day that *CCA1* peak expression occurs must shift by 4 h over the course of the year. This adjustment occurs because the oscillator can entrain to the new time of the dawn signal through phase adjustments each day^44^.

Changes in photoperiod also adjust the time of peak expression of the components of the oscillator (Fig. 2)^44^. As the day length increases, there is an increase in the circadian period^13^, which might be why the oscillator genes peak later after dawn in the long photoperiods (Fig. 2)^44^. While all oscillator genes tend to peak later in long photoperiods, the genes are not completely locked to each other in terms of phase. In an experiment in which the oscillator transcript abundance was measured across light and dark (diel) cycles with day lengths of 6 h or 18 h, *LHY* peaked 0.9 h later in the 18-h photoperiod compared to the 6-h photoperiod, whereas the timing of peak abundance of *TOC1/PRR1* was 3.7 h later in the longer photoperiod and *PRR9* was 4.5 h later^44^. If the components of the oscillator were tightly linked, they would respond similarly to different lengths of photoperiod. Instead the relationship between the components in terms of the time of peak expression appears to have a degree of plasticity. The consequence is the time between the activity of each of the components can change dramatically. For example, in the short photoperiod, the time between the peak of *CCA1* to *PRR9* is 4.3 h, whereas in the long photoperiod it is 6.1 h^44^. Similarly, in the short photoperiod, there is a 3.9-h gap between peak *PRR9* and *PRR7*, but in the long photoperiod this is reduced to 0.4 h. Some components appear to have tighter linkage with each other, such as those that form the evening complex (*ELF3*, *ELF4* and *LUX*), which peak nearly simultaneously in both photoperiods (Fig. 2).

In addition to the relative plasticity in the timing between some of the components, there is also a plastic relationship between the phase of the oscillator components and the timing of the light and dark cycle. While *CCA1/LHY* always peak near dawn, there is not a similar relationship between other oscillator genes and the light/dark cycle (Fig. 2). For example, in the short photoperiod, all the evening complex transcripts peak shortly after dusk, but in the long photoperiod, the peak is just after midday (Fig. 2). The time of peak *TOC1/PRR1* and *GI* expression also shifts from the dark to the light in the different photoperiods (Fig. 2).

While the timing of the abundance of the RNA molecules might not reflect completely the timing of the activity of the encoded proteins, the change in the timing of expression of the oscillator genes demonstrates regulatory plasticity in the oscillator because the expression of the oscillator transcripts represents the output of the regulatory events in the oscillator and shows the plastic response of these regulatory events to changes in photoperiod. This plasticity of the phase of the components of the circadian oscillator demonstrates that the genes should not be considered like cogs tightly coupled in a clock mechanism. The flexibility of the phase of peak expression is well captured by models that consider the system to be a complex regulatory network, rather than comprised of discrete feedback loops^45^. The proposal that the Arabidopsis circadian oscillator comprises coupled bistable systems also might in part explain how oscillatory dynamics can arise from a system in which the timing of the expression of the components is too plastic to consider the oscillator as a tightly coupled oscillator akin to a watch^43^.

### Dynamically plasticity might permit entrainment

**Fig. 3:**
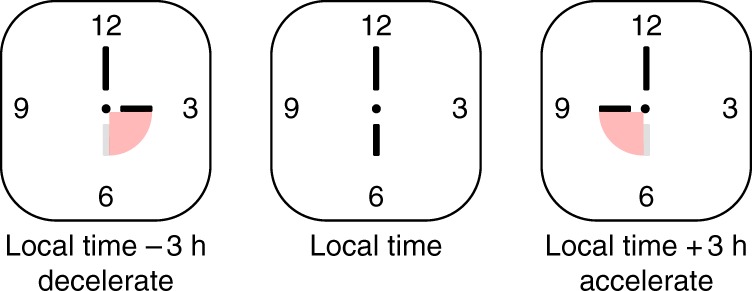
Entrainment might involve transient changes in the velocity of an oscillator. We can conceptualise the problem of entrainment as setting the hands of a travel clock when moving between time zones. In the cartoon, we can see that if we move the time zones and readjust the hands of the clock to a new time zone, there is a temporary speeding up or slowing down of the movement of the hands to adjust to the new time zone, before the clock returns to its fixed velocity

Synchronisation to the local environment is the essence of a circadian system and might be more important than the characteristic ability of circadian systems to generate self-sustained rhythms because plants that have functional oscillators perform poorly in environments in which they cannot entrain well to the light and dark cycle^2,46^. We can conceptualise the problem of circadian entrainment as the resetting of the time of a mechanical travel clock after jet flight to a new time zone^27^. The pace of the ticking of the clock will depend on the mechanical clock work, whereas the position of the hands, equivalent to the phase, will be set by the owner of the clock (Fig. 3). The owner will set the hands of the clock to the local time where they are based, with the owner being the equivalent to an entraining zeitgeber (time setting signal). Following a jet flight to a new time zone, the owner will adjust the hands of the clock to the new time zone by winding the mechanism clockwise or anti-clockwise, i.e. with or against the default rotation of the clock. It can be seen from this analogy, that the owner transiently changes the velocity of the rotation of the hands around the clock face. For example, a clockwise rotation of the hands from six to nine o’clock represents a transient acceleration of the velocity. Following the entraining change in velocity to the new position, the hands will subsequently return to the default free-running velocity determined by the clock mechanism. Below, we outline the evidence that stimulus-induced transient changes in circadian period are also associated with the entrainment of circadian oscillators. While it is important for circadian oscillators to respond to signals, these responses must not be unfettered. There are trade-offs in the architecture of circadian systems, because the most precise clocks, which are resistant to external perturbation, are the slowest to entrain, and therefore lower precision favours robust entrainment; however, a degree of precision is required to prevent instability^47^.

Mutations in *BIG (At3G02260)*, a collosin-like protein, affect the dynamic plasticity of circadian period and result in plants that entrain less well than wild-type^13^. BIG was found to be involved in the dynamic plasticity of circadian period because mutations alter the response to nicotinamide^13^. Nicotinamide increases the circadian period in all organisms tested, including Arabidopsis^18^, mouse^48^ and Ostreococcus^49^. Nicotinamide might regulate the circadian period through the inhibition of ADPR cyclase activity and Ca^2+^ signalling^18^, SIRTUINs^48^ or reduction of H3K4me3 accumulation^34^. Mutations in *BIG* render the Arabidopsis circadian oscillator oversensitive to nicotinamide such that treatment will increase the circadian period by about 3 h in the wild-type, but up to 6 h in mutant plants^13^. The defect in dynamic plasticity of the period is not restricted only to responses to nicotinamide, *big* mutants are also oversensitive to light, having faster running oscillators at low light intensities than wild-type plants^13^. Therefore, *big* mutants, like *prr7*, have defects in their ability to dynamically adjust the circadian period, and both these mutants have altered phase in light and dark cycles^13,16,24,41,42^, and in the case of *prr7* mutants, there is also a phase defect following entrainment to temperature cycles^50^. These data suggest that the dynamic adjustment of circadian period is associated with entrainment to both light and temperature^13,16,24,41,42,50^. Dynamic plasticity of circadian velocity, and therefore period, is probably associated with entrainment in other organisms as well. Modelling studies have shown that the properties of entrainment of the mouse circadian oscillator are accounted for if the velocity of the oscillator is considered as being in continuous adjustment^51^.

**Fig. 4:**
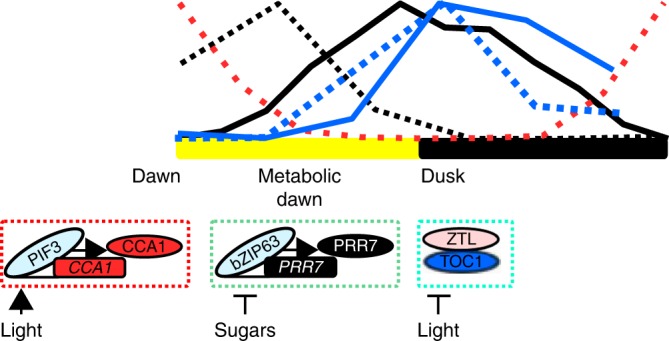
Dynamic phase adjustment of circadian oscillator through regulation of individual components. Entrainment occurs through signalling to individual components based on their temporal availability. At dawn, light-regulated transcription factors, including PIF3, regulate *CCA1* expression^33^, sugars regulate bZIP63 to modulate *PRR7* expression to establish a metabolic dawn^16,24^ and dusk is sensed through ZTL-mediated degradation of TOC1 protein in the dark^29^. Each entrainment event is semi-discrete, can have potential for downstream components, but might also be temporally restricted due to subsequent inputs. The sequential nature is akin to a signalling pathway rather than the resetting of an entire clock mechanism. The wave form for these components in a 12-h light and 12-h dark cycle normalised to peak height indicates the times of potential regulation. Red (CCA1), black (PRR7) and blue (TOC1). Dashed lines are mRNA, solid line is the protein. Data for the plots were obtained from digitised data sets^45^ of *CCA1* mRNA^71^, *PRR7* mRNA^72^ and protein^55^, and *TOC1* mRNA^39^ and protein^37^. This collection does not contain light/dark cycle data for CCA1 protein^45^

We can consider two plausible scenarios in which a change of circadian period is associated with entrainment. In one, a stimulus-induced change in the expression or activity of a component could change the time taken between one event in the oscillator and the next event (e.g. a change in the rate of transcription, translation or protein degradation), which would alter the velocity and therefore the period of the oscillator (Fig. 1c). For example, reducing the time between the production of one protein and the next will advance the phase because the velocity has increased, reducing period. Because oscillator components are active at different times of the day (Figs. 1, 2 and 4), a stimulus-induced alteration can be restricted to a specific part of the pathway and therefore temporary, which will mean there is a temporary change in velocity of the oscillator, representing dynamic plasticity. An example of a transient, stimulus-induced change in abundance of an oscillator component is sugar-induced repression of *PRR7* expression in the morning, which is sufficient to advance circadian phase^52^. This could be considered a form of parametric entrainment because the expression of *PRR7* can be modelled by a parameter describing the range of possible values of *PRR7* abundance dependent on sugar stimulus strength^52^. A second scenario describes the so-called non-parametric mechanisms. In this scenario, dynamic alterations of circadian period could be a consequence of discontinuous changes in phase, caused by a large and rapid change in the state of an oscillator component, which results in a “jump” to another phase without an apparent change in velocity (Fig. 1c). Jumps in phase could result from rapid stimulus-induced changes in the state of an oscillator protein, such as protein–protein interactions, phosphorylation or bistability in the network. While an instantaneous change in phase does not change the velocity, it will change the period, because the period is the velocity plus the phase change.

### Dynamic adjustment by the regulation of oscillator components

It is reasonable to consider how the circadian oscillator arrives at an appropriate period and phase when regulated by multiple inputs such as light and sugars. Possibly, light and sugars are competing signals, and the “strongest” signal is the one that determines the phase of the oscillator. However, because the components of the circadian oscillator are not rigidly locked to each other^44^, it is possible to consider an alternative scenario in which there might be multiple resetting events that dynamically adjust the phase, dependent on input signals regulating individual components of the oscillator at different times of day. We have proposed that in addition to dawn, there is a second phase setting event that occurs through the regulation of *PRR7* expression to adjust to a “metabolic dawn”^16^. In this model, there is a temporal separation between the regulation of *CCA1/LHY* by light at dawn and the regulation of *PRR7* by sugar signals at the metabolic dawn. Regulation by the signals is restricted to specific times of day, dependent on the temporal dynamics of the wave of expression of the circadian oscillator components (Fig. 4). Continuous application of sugars speeds up the oscillator (Table 1), whereas a pulse of sugar causes an advance in phase, which is restricted to early in the photoperiod because that is the time when *PRR7 is* expressed. *PRR7* is the first transcriptional response of the oscillator to sugars and *PRR7* is required for the response to sugars^16^. Sugars regulate *PRR7* abundance through the sugar-signalling transcription factor bZIP63, which is also required for the response of the circadian oscillator to sugars in the morning^24^. Mathematical modelling explains why suppression of *PRR7* expression causes a phase advance only in the morning and a phase delay if the sugar is applied at night; in the absence of sugars, *PRR7* transcripts accumulate in the day and are low at night. A sugar signal in the day suppresses *PRR7* transcript abundance advancing the oscillator to a more night-like phase, whereas if that sugar signal arrives at night, it delays the accumulation of *PRR7* delaying the onset of the day phase of the oscillator^52^. The importance of this adjustment of circadian phase by sugar-mediated regulation of *PRR7* through bZIP63 is demonstrated by the late phase of *prr7-11* and *bZIP63-1* mutants in light and dark cycles^24^.

It has been proposed that sugars also regulate the circadian oscillator through binding of PIFs to the promoters of *CCA1* and *LHY*^53^. While induction of *CCA1* expression by sugars is attenuated in *pif* mutants^53^, *CCA1* might not be the primary entry for sugar signalling into the circadian system because sugar-induced increases in *CCA1* promoter activity are absent in *prr7-11*^16^. Furthermore, *PRR7* transcripts are upregulated in the first cycle of treatment with the photosynthesis inhibitor DCMU (3-(3,4-dichlorophenyl)-1,1-dimethylurea), which causes low sugar availability, but *CCA1* expression does not change until the subsequent cycle, presumably as a consequence of the regulation of *CCA1* by PRR7^16^. Sugar-mediated dynamic regulation of circadian period and phase might apply also to other species because sugars are also potential regulators of the circadian oscillator of the Crassulacean acid metabolism (CAM) plant *Kalanchoë fedtschenkoi*^54^.

In a light and dark cycle, the effect of a signal, such as sugar, on the phase will persist only until another zeitgeber (time-giver) adjusts phase through another component of the circadian oscillator (Fig. 4). For example, in Arabidopsis, the light-to-dark transition at dusk might adjust phase through the regulation of TOC1, whose abundance in the dark is regulated by ZTL and GI^29^. Once the cycle proceeds to the next dawn, light activation of the *CCA1/LHY* promoters will result in another phase adjustment of the circadian oscillator^33^. Considering light regulation of *CCA1/LHY*, regulation of *PRR7* by sugar status and input of dusk through TOC1 stability, it is possible to see that there can be dynamic phase changes occurring through the cycle by impact on the activity of individual components, which do not necessarily feed through to the next diel cycle because later zeitgebers also adjust the oscillator (Fig. 4). Therefore, circadian phase is in constant dynamic adjustment. This becomes more apparent when it is considered that other signals adjust the phase of the circadian system of Arabidopsis, for example temperature can entrain the oscillator through mechanisms that include *PRR7* and *9* (refs. ^50,55,56^) and nitrogen status also can regulate the phase of the circadian clock by regulating *CCA1* expression^57^. This plasticity of phase offers the opportunity for fine tuning the timing of the components and their regulated outputs in an ever-changing environment.

### Dynamically plasticity contributes to carbon homoeostasis

**Fig. 5:**
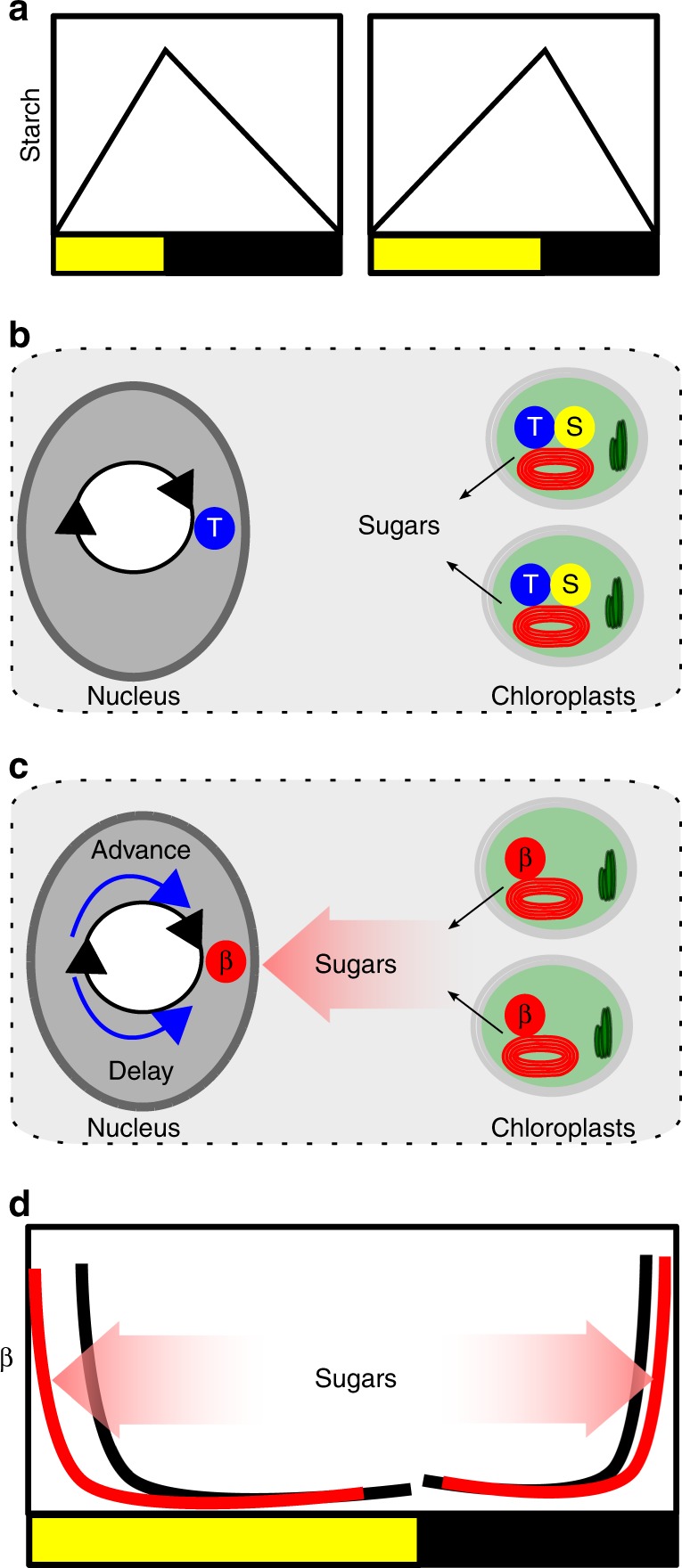
Dynamic plasticity of circadian rhythms might contribute to carbon homoeostasis in Arabidopsis. **a** There are diel changes in the transient starch pool stored in the chloroplasts, with the rate of accumulation and loss being near linear and adjusting to the photoperiod. **b** It has been proposed that the circadian clock generates an output that is a measure of time until dawn (T). This interacts with a measure of the size of the starch granule (S) to set a constant rate of starch degradation until dawn. In this model, the rate of degradation is calculated individually in the 100s of chloroplasts in a mesophyll cell. This model class assumes that starch pool size is regulated^63^. **c** Another proposal is that the circadian clock regulates the starch degradation activity (β), such that it peaks near dawn. Sugars released from starch degradation in the chloroplast feedback to regulate the circadian oscillator in the nucleus by affecting the pool of available C. The circadian oscillator is dynamically adjusted to maintain available C homoeostasis in a form of retrograde signalling^58^. **d** In the morning, a rising rate of change of sugars promotes phase advance (red), which will have the effect of reducing β at any particular time point. A phase delay in the morning will increase β at any time point, increasing starch degrading activity (not shown). Phase delays by sugars at night will have the effect of reducing β

Many of the hormones, metabolites and ions that regulate the period of the circadian oscillator (Table 1) do not have obvious associations with the timing of the dawn or dusk and are therefore unlikely to contribute to ensuring synchronisation of the circadian oscillator to external cues. More likely they provide feedback from the physiological and metabolic status of the plant to fine tune components of the circadian oscillator to ensure the oscillator regulates physiological and metabolic pathways appropriately. The “metabolic dawn” hypothesis is an example in which the oscillator is proposed to entrain to the varying rate of photosynthetic production of sugars dependent on prevailing weather conditions, possibly to adjust the timing of the production of transcripts and regulation of pathways involved in photosynthesis and metabolism^16^. Feedback from endogenous sugar levels to the circadian oscillator was shown by the altered circadian period and phase in low CO_2_, or following treatment with the inhibitor of photosynthesis, DCMU^16^. An extension of this concept proposed that the circadian oscillator might contribute to the homoeostatic management of transient carbon reserves associated with the light and dark of the diel cycle^58,59^. C3 and C4 plants fix carbon (C) during the day by photosynthesis. A percentage of the fixed C is allocated to a C reserve for consumption at night to survive the dark. In Arabidopsis, the transitory reserve is starch, which is consumed completely by dawn, with the rate of accumulation and loss being dependent on day length and near linear (Fig. 5a)^60^. The circadian clock is a regulator of the starch reserve, and if this regulation is compromised, there is a severe impact on growth^61^.

The transitory starch reserve is stored in the chloroplast as polymers of glucose highly compacted into a granular structure. The network of reactions involved in its synthesis and degradation is not a linear pathway, which has rendered the entire process a challenge to untangle. Degradation involves reversible phosphorylation of the glucan chains at the surface of the granule to disrupt its semi-crystalline structure. This enables hydration for the cleavage of α-(1,4) glycosidic bonds by multiple isoforms of α- or β-amylase, releasing malto-oligosaccharides or free maltose, respectively. Maltotriose residues are further metabolised into glucose that together with maltose are exported to the cytosol across the chloroplast inner envelope^62^.

To resolve how this remarkable process can adjust to day length, mathematical models of possible mechanisms have been proposed. One suggestion is that the plant calculates the nocturnal starch degradation rate based on the amount of starch at dusk and the time remaining to dawn, estimated by the circadian oscillator^63^. In this model, the circadian oscillator is a timekeeper providing an output of circadian time (T). This has been envisaged as two molecules, T (circadian output) and S (starch measure), which interact on the surface of the starch molecule to regulate the nocturnal degradation rate (Fig. 5b). The assumption that a value associated with the amount of starch at dusk is measured, setting a fixed degradation rate to dawn, has a strong basis in experimental observation; the starch degradation rate at night increases with the intensity of light during the day to ensure that the extra starch accumulated by dusk is exhausted by dawn and starch degradation rates also adjust to the imposition of an unexpected late or early dusk^64^. Although the identity of S and T are unknown, it has been speculated that the phosphorylation and dephosphorylation cycle at the starch surface could be a good candidate for controlling the starch dynamics. This hypothesis is supported by experiments showing that mutants of phosphoglucan water dikinase (PWD), which phosphorylate the C3-positions of glucose, are unable to correctly adjust starch degradation to an unexpectedly early night, and the level of phosphate per unit mass of starch along a diel cycle resembles the predicted behaviour of the S molecule concentration^63^. The model has been expanded to consider the potentially different roles of light-, dark- and circadian-regulation of starch degradation and synthesis^64^. It has been proposed that the stress-signalling kinase AKINβ1 is a good candidate for a dark sensor to allow adjustment of starch degradation rate to photoperiod^65^.

In an alternative model, starch is not measured. Instead a molecule, or signal, that reflects the available C-status (from photosynthesis and released by starch degradation) adjusts the phase of a circadian-regulated starch degrading activity (β; Fig. 5c)^59^. The measured available C pool is the C that is not in a storage pool such as starch and instead is the pool that is available for allocation to respiration, growth or storage. In the model, this pool was described as “sucrose” to distinguish it from starch but recognises that sucrose represents only a subset of the available C pool and might not be the sugar that is measured. Here, we will refer to homoeostasis of available C, unless the effects of specific sugars are being described. β could represent the activity of one or several starch degrading enzymes, which increase activity through the night to peak near dawn and decrease activity to a trough near dusk (Fig. 5d). β ensures that sufficient sugars are released to maintain available C homoeostasis. A first version of this model considered the starch degrading activity (β) as a simple sinusoidal that adjusted phase in response to available C status and was optimised to minimise starvation of the plant^66^. This was later revised to consider that the homoeostasis of available C is optimised, rather than starvation, because starvation rarely occurs in normal conditions^58^. In these models, an aspect of the circadian system is dynamically adjusted to regulate the phase of β and starch is not measured.

*prr7-11*, which is unable to dynamically adjust the circadian oscillator in response to sugars and other signals, has a higher rate of starch accumulation in long days than wild-type, providing empirical support for the hypothesis that the dynamic adjustment of the circadian oscillator contributes to the timing of starch degradation^58^. The proposed role of the dynamic adjustment of the oscillator by available C is most easily understood in simulations and experiments with a transfer between day/night cycles of different photoperiod lengths because this perturbs C homoeostasis. For example, if a plant is transferred from 16 h light/8 h dark to 8 h light/16 h dark, the nocturnal starch degradation is insufficient to support available C homoeostasis during the first night because insufficient starch has been accumulated and the night is long, and thus at dawn available C levels are low. When photosynthesis starts, there is a large positive-going available C signal advancing the circadian phase, which reduces β at each time point in the morning (Fig. 5d). This lower β during the photoperiod results in more starch accumulation (because less starch is being degraded) ensuring enough starch is stored to allow for C homoeostasis in the subsequent long night. Conversely, transfer from a short day to a long day results in excess available C at night, which results in a negative-going available C signal at dawn, and delay of circadian phase. The prediction from these simulations is that a circadian oscillator that cannot dynamically adjust the timing of starch degradation in response to the rate of change of available C will have higher starch accumulation in long days because the system will not respond appropriately to the dawn available C signals, which was confirmed experimentally in *prr7-11*^58^.

The dynamic adjustment of the circadian oscillator to available C is proposed to be most important in regulating the starch degrading activity during the day, rather than at night, since the circadian phase dependence of the night time starch degrading activity required to maintain available C homoeostasis is almost invariable regardless of the photoperiod^58^. This model explains why sugars advance the circadian phase in the morning and cause a delay in phase at night^16^, by demonstrating that minimisation of available C fluctuations in response to a simulated pulse of sugars in the morning requires an advance of the circadian phase of β to a point on the curve where there is a lower level of activity to reduce the amount of sugars released from starch. At night lowering β to cope with a pulse of sugar requires a phase delay (Fig. 5d)^58^.

It is probable that sucrose is not measured, but instead trehalose-6-phosphate (T6P), a signalling sugar, is the marker because in addition to adjusting the phase of the circadian oscillator^24^, T6P regulates the rate of starch degradation in the dark^67^. Low C-induced upregulation of *PRR7* expression by bZIP63 is repressed by T6P modulation of KIN10 activity, suggesting a signalling pathway by which the circadian oscillator adjusts the phase to regulate β^24^. However, until a candidate for β is identified, it will be difficult to distinguish whether T6P directly regulates starch degradation activity^67^ or regulates starch degradation through modulation of the circadian phase^24^, or both.

The two model classes proposed for the regulation of transitory starch pools (Fig. 5) differ not only in the proposed roles of the circadian oscillator, but also in considering the systems as examples of anterograde (nuclear to organelle; Fig. 5b) or retrograde (organelle to nuclear; Fig. 5c) signalling^68^. In the anterograde pathway, the nucleus distributes a timing signal T to each of the starch granules in each of the 100s of chloroplasts of a mesophyll cell to regulate the degradation of the starch (Fig. 5b)^63^. In the retrograde pathway, changes in available C due to sugars released from the degradation of starch feedback to the nucleus to regulate the circadian control of starch degradation offering the advantage of centralised regulation of distributed stores, in this case nuclear control of starch granules in 100s of chloroplasts (Fig. 5c). The need to regulate numerous stores suggests it is likely that some form of feedback must participate in the pathway, and this appears to involve the regulation of starch degradation by T6P, with current evidence being both for^24^ and against^67^ the circadian clock participating in this feedback.

Cereals accumulate transient starch in the leaf in a linear manner, similar to Arabidopsis, but usually the major transient carbohydrate is sucrose stored in the vacuole^69^. Understanding why some species store sucrose rather than starch, and the mechanisms by which this occurs will provide regulatory insight. The linear accumulation and storage of carbohydrate stores is not limited only to plants. Glycogen storage in Cyanobacteria has similar linearity of accumulation and loss, dependency on photoperiod for the rate of accumulation and involves circadian regulation^70^. As with starch in Arabidopsis, it has proven possible to model the timing of glycogen accumulation as an emergent property of optimal resource allocation on the basis of growth optimisation^58,70^. Other forms of homoeostasis might also be associated with the dynamic adjustment of the circadian oscillators because temperature compensation of the Arabidopsis circadian oscillator involves the regulation of SUMOylation, and mutants that are defective in this process have altered dynamic changes in period in response to temperature^15^.

### Circadian oscillators are not uniquely isolated from biology

The new data demonstrating a role for signals in regulating the period and phase of the circadian clock in Arabidopsis show that the plant circadian oscillator is not ticking away invariantly isolated from the rest of the cell. Instead circadian period and phase are dynamically adjusted (Table 1). The components of the oscillator are not locked together like the cogs of a watch, they can respond semi-independently to the different signals, for example *PRR7* is upregulated by low sugar signals, whereas *PRR9* is unresponsive^16^ and the oscillator components change their temporal relationship in response to changes in the photoperiod^44^. These findings suggest that the circadian oscillator is a dynamically adjusting system that responds to many signals to participate in synchronising internal time with external time, and possibly to regulate the biology of the cell.
